# Correction to: Estimates of the permeability of extra-cellular pathways through the astrocyte endfoot sheath

**DOI:** 10.1186/s12987-023-00456-x

**Published:** 2023-07-26

**Authors:** Timo Koch, Vegard Vinje, Kent‑André Mardal

**Affiliations:** 1 Department of Mathematics, University of Oslo, Postboks 1053 Blindern, 0316 Oslo, Norway; 2 Simula Research Laboratory, Kristian Augusts gate 23, 0164 Oslo, Norway

**Correction:** ***Fluids Barriers CNS*** **20, 20 (2023)**

10.1186/s12987-023-00421-8

Following publication of this article [1], the author group became aware of some errors in the Results section of the Abstract.

These errors were introduced during typesetting.

The correct values are given below and the original article has been corrected.

The publisher would like to apologise for any inconvenience caused.

## Results

We provide structural-functional relationships between vessel radius and resistance that can be directly used in flow and transport simulations. We estimate endfoot sheath filtration coefficients in the range L_p_ = 2 × 10^− 11^ m Pa^− 1^ s ^− 1^ to 3 × 10^− 10^ m Pa^− 1^ s ^− 1^, diffusion membrane coefficients for small solutes in the range C_M_ = 5 × 10^2^ m^− 1^ to 6 × 10^3^ m^− 1^ and gap area fractions in the range 0.2–0.6%, based on a inter-endfoot gap width of 20 nm.
